# The Delphi Method: Developing a Telerehabilitation Practice Guideline for Patients in Indonesia with Long COVID

**DOI:** 10.5195/ijt.2024.6610

**Published:** 2024-06-28

**Authors:** Nurul Paramita, Dewi Irawati Soeria Santoso, Nury Nusdwinuringtyas, Menaldi Rasmin, Neng Tine Kartinah, Sri Widia A. Jusman, Murdani Abdullah, Damayanti Tinduh, Siti Chandra Widjanantie, Melinda Harini, Imelda Rosalyn Sianipar, Boya Nugraha, Christoph Gutenbrunner, Sandra Widaty

**Affiliations:** 1 Doctoral Program in Medical Sciences, Faculty of Medicine Universitas Indonesia, Jakarta, Indonesia; 2 Department of Medical Physiology and Biophysics, Faculty of Medicine Universitas Indonesia, Jakarta, Indonesia; 3 Department of Physical Medicine and Rehabilitation, Faculty of Medicine Universitas Indonesia, Dr. Cipto Mangunkusumo Hospital, Jakarta, Indonesia; 4 Department of Pulmonology and Respiratory Medicine, Persahabatan National Respiratory Referral Hospital, Faculty of Medicine Universitas Indonesia, Jakarta, Indonesia; 5 Department of Biochemistry and Molecular Biology, Faculty of Medicine Universitas Indonesia, Jakarta, Indonesia; 6 Department of Internal Medicine, Faculty of Medicine Universitas Indonesia, Dr. Cipto Mangunkusumo Hospital, Jakarta, Indonesia; 7 Faculty of Medicine, Airlangga University, Surabaya, Indonesia; 8 Department of Physical Medicine and Medical Rehabilitation, Dr. Soetomo General Hospital, Surabaya, Indonesia; 9 Department of Physical Medicine and Rehabilitation, Persahabatan National Respiratory Referral Hospital, Faculty of Medicine Universitas Indonesia, Jakarta, Indonesia; 10 Department of Rehabilitation and Sport Medicine, Hannover Medical School, 30625 Hannover, Germany; 11 Hannover Rehabilitation Services & Science Consulting, 30627 Hannover, Germany; 12 Department of Dermatology and Venereology, Faculty of Medicine Universitas Indonesia, Dr. Cipto Mangunkusumo Hospital, Jakarta, Indonesia; 13 Department of Medical Education, Faculty of Medicine Universitas Indonesia, Dr. Cipto Mangunkusumo Hospital, Jakarta, Indonesia

**Keywords:** Delphi method, Guideline, Long COVID, Rehabilitation, Telerehabilitation

## Abstract

Telerehabilitation has the potential to help expand the reach of rehabilitation intervention. An online questionnaire-based Delphi method set out to develop a telerehabilitation guideline for patients in Indonesia with Long COVID. A Delphi panel comprised of 24 experts was selected from all relevant disciplines. Over two rounds of Delphi testing, panelists gave opinions and indicated their level of agreement with each recommendation. Key elements of consensus for a telerehabilitation guideline for patients with Long COVID includes: the benefit of telerehabilitation, types of rehabilitation intervention needed, methods of intervention, criteria for home-based self-exercise training, set-up of rehabilitation prescription, exercise monitoring, evaluation of rehabilitation intervention and duration of rehabilitation intervention. Further research is needed to determine the feasibility and effectiveness of this guideline.

After the COVID-19 pandemic it became evident that for a considerable number of patients, clinical symptoms may last beyond the acute phase, and a complex multisystem illness could ensue either immediately or sometime after apparent recovery from the acute phase, irrespective of the severity (Datta et al., 2020; Nalbandian et al., 2021). Studies have revealed that Long COVID follows even mild to moderate cases, even in patients who did not require respiratory support, intensive care, or hospitalization (Dennis et al., 2021; Townsend et al., 2021).

Various terms are used to express this condition, including “Long COVID,” “post COVID-19 conditions,” and many others. According to the National Institute for Health and Care Excellence the term “Long COVID” is commonly used to describe signs and symptoms that continue or develop after acute COVID-19. The term includes both ongoing symptomatic COVID-19 (from 4 to 12 weeks) and post-COVID-19 syndrome (12 weeks or more)(NICE et al., 2020). In October 2021, the World Health Organization (WHO) published a working clinical case definition of post-COVID conditions. The WHO characterized post COVID-19 as symptoms that occur in persons with a history of probable or confirmed SARS-CoV-2 infection, usually 3 months from the onset of COVID-19, with symptoms persisting no less than 2 months. Symptoms may be of new onset following initial recovery from acute COVID-19 or persisting from the initial illness. Symptoms may also fluctuate or relapse over time. (Soriano et al., 2022) and cannot be justified by another diagnosis.

There is no definitive data regarding the incidence of Long COVID. As of March 2023, it was estimated that 1.8 million people living in the UK (2.8% of the population) were experiencing self-reported Long COVID (Office for National Statistics, 2023). Data from the U.S. Centers for Disease Control and Prevention (CDC) stated that overall, 1 of 13 adults in the U.S. (7.5%) have Long COVID symptoms (CDC, 2022). A 6-month follow-up during September and November 2022 showed that out of 21.797 COVID-19 patients discharged from Huashan Hospital, China, 8.89% had Long COVID symptoms (Cai et al., 2023). In Indonesia, during the first wave of COVID-19, a cross-sectional study was conducted using an online questionnaire from 9 to 28 January 2021. Of 385 respondents, 256 (66.5%) respondents developed prolonged COVID-19 symptoms, and 16.8% reported persistent symptoms for more than three months (Susanto et al., 2022). No recent data is available for the prevalence of Long COVID in Indonesia.

Long COVID presents as subjective symptoms and impacts quality of life and mental status. One study showed that problems with mobility, personal care, and activity were prevalent in patients with Long COVID. Fatigue, sleep difficulties, and cough were among the most reported symptoms (Cai et al., 2023). A survey conducted on 3,762 COVID-19 survivors from 56 countries revealed that many people with Long COVID experienced decreased work capacity relative to pre-illness levels. On average, this group reported less than 60% of their pre-illness levels (Davis et al., 2021). Another study on COVID-19 survivors who experienced Long COVID showed that COVID-19 decrease their energy reserves (Humphreys et al., 2021). Schmachtenberg, et al. (2023), conducted a guided interview of 25 people with Long COVID and concluded that people with Long COVID face social limitations that impair their daily activities, personal interests, and occupational life. The inability to work, failed attempts to return to work, and continuing limitations after vocational reintegration cause despair for the patients (Schmachtenberg et al.). One survey showed that people who experience Long COVID needed better support to manage their symptoms, especially fatigue, and needed assistance to safely experience the potential benefits of physical activity. Survey participants reported that guidelines on physical activity were not always adapted to the complexity of Long COVID (Humphreys et al., 2021).

Many studies have been conducted to assess the impact and benefits of rehabilitation interventions on an illness. The rehabilitation intervention is given in a structured manner and usually includes the provision of a physical exercise program. The results of these studies show improvements in aerobic capacity, functional ability, and quality of life (Blondeel et al., 2018; Morris & Chen, 2019; Simonelli et al., 2019). Gutenbrunner et al. (2020) emphasize the importance of rehabilitation interventions as an integral part of the management of COVID-19 patients from the acute, post-acute, and Long-term phases, all the while still paying attention to patient safety. Long-term rehabilitation services must provide multimodal, patient-centered rehabilitation which aims to return the patients to their pre-illness state. Such rehabilitation services should be carried out by a multi-professional team (Gutenbrunner et al.).

The National Institute for Health and Care Excellence (NICE), the Scottish Intercollegiate Guidelines Network, and the Royal College of General Practitioners have developed a rapid guideline to manage Long COVID and establish Long COVID services. The guideline is a “living guideline” that is updated regularly as new evidence emerges. Rehabilitation practice is included in the guideline as part of the management for Long COVID. Such rehabilitation practice may include providing information, education, supported self-management, peer support, symptom management strategies and physical rehabilitation. Given the current scarcity of supporting evidence, the guideline still lacks detail on potentially helpful rehabilitation interventions, but it emphasizes comprehensive assessment by multidisciplinary team and support the use of telerehabilitation (NICE et al., 2020).

Telerehabilitation is a part of telemedicine that is still developing. While research on the use of telerehabilitation is ongoing, many studies have a limited number of subjects. However, studies have shown that telerehabilitation has the potential to help expand the reach of rehabilitation programs (Peretti et al., 2017). One systematic review and meta-analysis in patients with heart failure showed that telerehabilitation contributes to a better quality of life due to the daily increase in social activities, exercise tolerance, mental health, and improvement of symptoms such as edema, fatigue, and dyspnea (Cordeiro et al., 2022). A short-term, supervised virtual telerehabilitation program based in India has improved walk test performance, daily ambulation, and health-related quality of life (HRQoL) in cardiac, pulmonary, and oncology patients (Patel et al., 2021).

In Indonesia, telemedicine start-ups began to become popular after the COVID-19 pandemic (Ritmeester, 2020). In their systematic study, Nugraha et al. (2020) highlighted the status and challenges of physical medicine and rehabilitation intervention in Indonesia due to the COVID-19 pandemic. They concluded that at the hospital level, it is very important to ensure the availability of COVID-19 rehabilitation services for all phases (acute and chronic), both for inpatients and outpatients. One recommendation was to apply telerehabilitation practice as a supportive and complementary treatment for patients (Nugraha et al.). It is expected that telerehabilitation could expand the reach of rehabilitation intervention and improve patient's compliance to the rehabilitation program by reducing the needs of in-person meetings between health workers and patients with Long COVID.

Reis et al. (2023), explored the pre-post effects of telerehabilitation (i.e., a home-based rehabilitation program supervised by video calls) in patients with Long COVID in control and intervention groups. The intervention group received an intensive rehabilitation program three times a week for 12 weeks followed by a maintenance rehabilitation phase once a week for 2 weeks. The control group received the usual care. Post-intervention, as compared to the control group, the intervention group presented with a lower heart rate (p = 0,005); better post COVID functionality (PCFS) (p < 0,0001); improvement in fatigue (p < 0,0001); reduced dyspnea (p < 0,0001); ability to perform personal care (p = 0,034); ability in leisure activities (p = 0,01); improvement in the Sit to Stand Test (p < 0,0001); and decreased anxiety (p = 0,004) and depression (p < 0,0001)(Reis et al.)

A study by Calvo-Paniagua et al. (2022), using a quasi-experimental design, also explored the result of a telerehabilitation practice in 71 patients with Long COVID. The telerehabilitation consisted of patient education, physical activity, airway clearing, and breathing exercise interventions for eighteen sessions (3 sessions/week). The exercise-based rehabilitation practice was performed in a telehealth modality by video conference using Zoom. Compared to baseline, the distance walked during 6-minute walk test was significantly greater after finishing the intervention (p < 0,001; η^2^p = 0.065), after 1 month and after 3 months (all, p < 0,001). Daily living activities, dyspnea severity, and quality of life improved significantly post intervention and at all follow-ups (all, p < 0,001)(Calvo-Paniagua et al.).

One randomized clinical trial analyzed the clinical efficacy of telerehabilitation intervention in the recovery of patients with Long COVID using a mobile app (ReCOVery APP). The control group followed the usual treatment established by their general practitioner. The intervention group followed the same methods with the additional use of ReCOVery APP. The analysis comparing the pre-intervention and 3-month post-intervention showed no significant difference between the control and intervention groups; however, a linear regression model predicted that the time of use of ReCOVery APP increased physical functioning (p = 0,005 CI 95% 0,000 – 0,002) and improved community social support (p = 0,021 CI 95% 0,001 – 0,008)(Samper-Pardo et al., 2023).

The rehabilitation practice for Long COVID has been challenging because there are few randomized controlled clinical trials for Long COVID telerehabilitation intervention. There is an incomplete understanding of the pathophysiology of Long COVID, and variations in individual symptoms.

In this study, we undertook an investigation of experts' experience and knowledge in Long COVID and telerehabilitation using the Delphi method. The Delphi method is an iterative process used to collect and distill the judgments of experts using a series of questionnaires interspersed with feedback (Boulkedid et al., 2011). The Delphi method elicits qualitative data (Skulmoski et al., 2007). It is a formal consensus development method which is considered for use if there is limited quantitative data (Boulkedid et al., 2011; Vernon, 2009). We assessed the expert agreement that could be used for provisional guidance about the benefits, types of precautions, and duration of telerehabilitation practice for patients with Long COVID that applies to conditions in Indonesia. This guideline is also intended to guide all rehabilitation teams who are providing telerehabilitation practice for patients with Long COVID.

## Method

### Expert Panel Selection

The present study was conducted in accordance with the Declaration of Helsinki and ethical guidelines regarding clinical research. Ethical approval for the current study was obtained from the Faculty of Medicine, Universitas Indonesia Research Ethics Committee (protocol number: 21-07-0713).

The research team met to discuss and determine the criteria and the number of multi-professional panelists. Taking into consideration that the aim of this study was to formulate guidelines for telerehabilitation practice, it was determined that a significant number of panelists must be doctors who specialized in physical medicine and rehabilitation (physiatrist). As the rehabilitation practice would be assisted by physiotherapists, it was determined that some of the panelists should be physiotherapists. Because the telerehabilitation guidelines would be specific for patients with Long COVID patients, the symptoms of Long COVID are varied, and existing management recommendations require a multidisciplinary approach, it was decided that some of the panelists should be pulmonologists, cardiologists, and neurologists. Given that rehabilitation interventions are to correspond to physical exercise prescriptions and Long COVID symptoms are closely related to physical activity tolerance, it was agreed that some of the panelists should be experts in the field of exercise physiology and sports medicine.

There is no standard concerning the appropriate number of panelists for the Delphi method. The number of panelists from prior Delphi studies varies greatly, ranging from three to more than 100 in multicenter and international studies (Niederberger & Spranger, 2020). Murphy et al. (1998) in the systematic review about the Delphi method states that approximately 16 to 28 panelists will be able to produce a joint consensus regarding the quality of care for a single case with a reliability of 0.95. Based upon the agreed criteria for this study, we decided to have 24 panelists consisting of five physical medicine and rehabilitation specialists, three physiotherapists, three pulmonologists, three cardiologists, three neurologists, three sports medicine experts, and three exercise physiologists.

### Survey Process

To develop the guideline, all authors first discussed the basic assumptions of this guideline based on literature reviews and their own experience. As a result of these discussions, the first author generated statements that aligned with the framework. All authors then discussed the appropriateness and coverage of the statements to reach a consensus.

**Figure 1:**
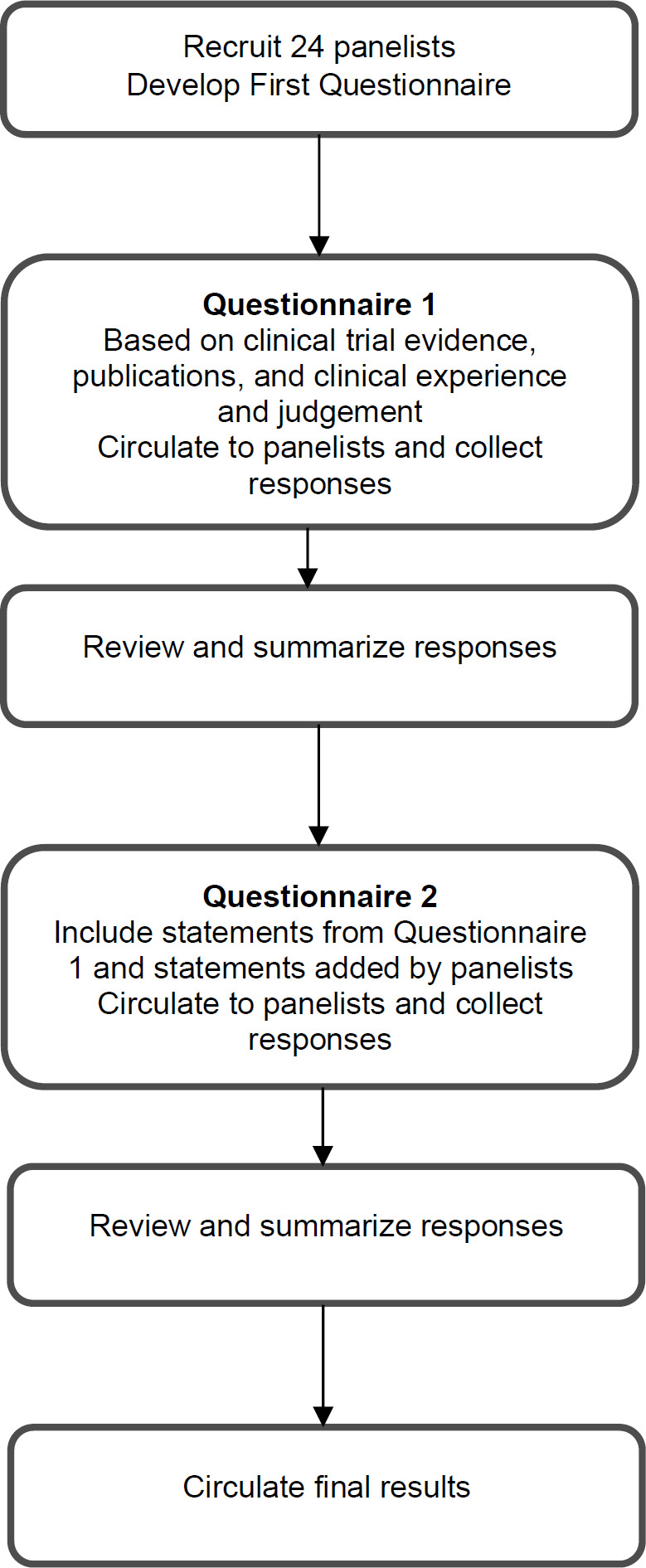
The Modified Delphi Process Used in This Study

The study was conducted from June 2022 to September 2022. A modified Delphi process (Figure 1) was designed in two rounds of questionnaires, emailed to panelists in sequence. Each round lasted five weeks and was separated by three weeks. All respondents were sent weekly text message reminders. No financial incentives were provided

### Delphi Round 1

An invitation e-mail, containing a URL link to the survey, was sent to the identified potential panelists with a request to respond within five working days. Upon clicking the link, the panelists were each asked to provide their contact information (name, email address), qualifications (specialty and eligibility criteria), baseline characteristics, and consent to participate in the Delphi study.

The panelists were requested to read a brief introduction of the background study and study processes, and to indicate their degree of approval for each of the 37 initial items on a five-point Likert scale (1 = ‘strongly agree', 2 = ‘agree', 3 = ‘neither agree nor disagree', 4 = ‘disagree', 5 = ‘strongly disagree'). They were also encouraged to comment on each item in a free-text box, particularly if there was any disagreement with any of the items. Six open ended questions were added to be answered by the panelists. Refer to Appendix A for the items presented in Delphi Round 1.

Item scoring and comments were downloaded, anonymized, and summarized by the first author. A discussion by all authors then facilitated review and revision of the items. For the Likert scale items, content validity ratio (CVR) of each item was determined and items eliciting a response of ‘strongly agree’ and ‘agree', of 0.417 or more was determined as showing consensus. The formula was CVR = (Ne – N/2)/(N/2), in which the Ne was the number of panelists indicating “essential” and N was the total number of panelists (Zamanzadeh et al., 2015). Items eliciting ‘strongly agree’ and ‘agree’ were considered essential. The cut off point for CVR was determined by Lawshe Table (Ayre & Scally, 2014).

Items with consensus were subject to minor adjustment when needed. If more substantial amendments were needed, the item was reworked based on panel comments and re-tested in Round 2, alongside items that did not obtain consensus initially. For open ended questions, all the responses were summarized and arranged into 5-point Likert scale questions to be tested in Round 2.

### Delphi Round 2

All panelists who took part in Round 1 were sent an email containing a URL link to the second questionnaire (Round 2), with a request to respond within five working days. Refer to Appendix B for the items presented in Delphi Round 2.

As in Round 1, level of agreement with sequentially presented items (either amended from Round 1 or newly added to Round 2) were required. In five items, panelists were asked whether they agree or disagree to the statements provided. In the rest of the 59 items, panelists were asked to designate whether an intervention “must be given,” “should be given in certain condition only,” or “no need to be given.” As in Round 1, the panelists were asked to type comments, including a reason should they disagree or think that an intervention would not be needed, as well as any additional opinions.

Item scoring and comments were downloaded, anonymized, and summarized by the first author. All authors reviewed and discussed the items. A Content Validity Ratio (CVR) was determined for each item. The formula applied was: CVR = (Ne – N/2)/(N/2), in which the Ne was the number of panelists indicating “essential,” and N was the total number of panelists (Zamanzadeh et al., 2015). At the initial stage, items eliciting “agree” (in items with the option of agree/disagree) and ‘must be given’ or ‘should be given in certain condition only’ (in the rest of the items) was considered essential. In this step, the option ‘must be given’ was considered essential. The next step was to determine whether there was agreement between “must be given” (positive agreement) or “should be given in certain condition only” (negative agreement). The cut off point for CVR was determined by the Lawshe Table (Ayre & Scally, 2014).

## Results

### Participant Characteristics

We contacted 24 eligible panelist candidates from 12 different hospitals and/or medical education institutions in Java and Sumatra Island through email and text messages. All respondents confirmed their willingness to participate in this study.

**Figure 2:**
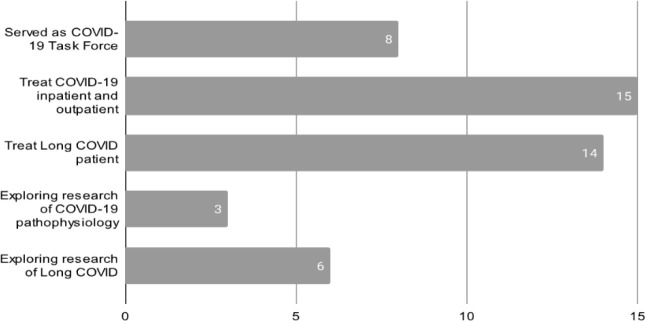
Characteristic of Panelists

Twenty-four Indonesian health practitioners, representing a wide range of characteristics (Figure 2), responded to the text message, and were emailed a link to the Round 1 questionnaire. Eight (25%) served as COVID-19 task force members and fifteen (62.5%) treated COVID-19 patients. All contacted panelists completed both Delphi Round 1 and Round 2.

### Delphi Round 1

The first round of the survey was performed from 13 June to 23 July 2022. All the 24 panelists responded and returned the questionnaire. The first-round questionnaire consisted of 37 statements. (See Appendix A). In the first-round survey, 34 of 37 (91.9%) statements were agreed upon by more than 70% of participants, and only three (8.1%) statements led to disagreements.

During the authors' discussions, all statements were carefully examined. We analyzed the reasons for the three disagreements and decided to revise those statements in the Round 2 questionnaire. We also analyzed comments about the 34 statements and decided to incorporate all of these statements into a Round 2 questionnaire, adding more specific options. In the first round, we had several open-ended questions. The questions elicited opinions regarding aspects of health that can be improved through rehabilitation practice; the essential rehabilitation practice; things that need to be assessed to determine rehabilitation prescriptions for patients with Long COVID; the criteria needed for Long COVID patients to be able to carry out physical exercise independently at home; concerns regarding the preparation of a telerehabilitation practice for patients with Long COVID. We listed, analyzed, and summarized answers from the panelists. For the Round 2 questionnaire, we incorporated all the answers into several closed questions. After the authors' discussion, a summary of the outcomes and a revised version of the guideline were sent to all panelists to confirm corrections and determine whether there were additional opinions.

### Delphi Round 2

The second-round of the survey was conducted from 4 August – 22 September 2022. All the 24 panelists responded and returned the questionnaire (See Appendix B). In this round, a list of 64 statements was arranged into 8 categories: benefit of rehabilitation practice; type and method of telerehabilitation practice; criteria for self-exercise training; set-up of the rehabilitation prescription; exercise monitoring; evaluation of rehabilitation practice; and duration of rehabilitation practice. Of the five agree or disagree statements, three were agreed upon by all panelists (100%) and two were agreed by 87.5% of the panelists. Of the next 59 items, there were three items that had 92% agreement, six items with 95.8% agreement, and 100% agreement for the rest of the items.

## Discussion

Key concepts for telerehabilitation in patients with Long COVID emerged from the Delphi process. Most on the panel agreed that providing rehabilitation practice to patients with Long COVID can provide physical, psychological, and social benefits. Table 1 shows opinions aspects that can be improved through rehabilitation practice.

**Table 1:** Aspects That Can Be Improved Through Rehabilitation Intervention in Patients with Long COVID

List of aspects
Cardiovascular function (including cardiovascular fitness level and exercise tolerance) Respiratory function (including dyspnea, short breath, and thorax expansion) Neuromusculoskeletal function (including muscle strength, muscle endurance, flexibility, coordination, and balance) Functional capacity Fatigue Metabolic condition Pain (headache, muscle pain, joint pain) Quality of sleep Psychological condition (stress, anxiety, depression) Cognitive function (including confusion and attention deficit) Independence Activity and participation (social life) Quality of life

Some of the aspects mentioned in Table 1 were already demonstrated in other recent studies with small sample sizes. Huang et al. (2022) performed a metanalysis to explore the benefits of telerehabilitation practice in patients with or survivors of COVID-19. That study showed the superiority of telerehabilitation over no treatment or usual care for dyspnea (Borg scale: mean difference = −1.88, −2.37 to −1.39; Multidimensional dyspnea-12: mean difference = −3.70, −5.93 to −1.48), limb muscle strength (mean difference = 3.29; 2.12 to 4.47), ambulation capacity (standardized mean difference = 0.88; 0.62 to 1.14), and depression (mean difference = −5.68; −8.62 to −2.74). No severe adverse events were reported in any of the included studies (Huang et al).

Because until recently the pathophysiology of Long COVID was not fully understood, conclusions of the benefits of rehabilitation practice for Long COVID were drawn from the benefits of rehabilitation practice and physical exercise in general. Evidence from previous studies have shown that the benefits of physical exercise include both physical and psychological aspects (Chodzko-Zajko et al., 2009). With appropriate frequency and intensity, regular exercise can induce lower heart rate at rest and during submaximal exercise, a smaller increase in blood pressure, increase in glucose transporter content in muscle, improved whole-body insulin action, and reduced plasma lipid concentration (Chodzko-Zajko et al., 2009). From epidemiological studies it was shown that regular physical activity can help prevent anxiety and depression symptoms (Pasco et al., 2011). One study showed that more physically fit and physically active subjects had fewer symptoms of depression (Galper et al., 2006). A systematic review and meta-analysis of randomized controlled trials has shown that exercise is both safe and beneficial for physical and psychosocial health in people with multimorbidity (Bricca et al., 2020). Rehabilitation and exercise training has been proven to improve functional capacity, reduce symptoms, and increase quality of life in patients with COPD, cardiovascular disease, and osteoarthritis (Fiuza-Luces et al., 2018; Gloeckl et al., 2018; Goh et al., 2019). Physical training correlates with improvement in cognition and delayed progression of Alzheimer's disease (De la Rosa et al., 2020).

Telerehabilitation as part of telemedicine has become necessary as a result of the COVID-19 pandemic. Since then, many rehabilitation professionals have integrated telerehabilitation into their rehabilitation services. In this study, regarding telerehabilitation practice for patient with Long COVID, all panelists agreed that rehabilitation could be implemented through telemedicine (telerehabilitation) with a few caveats as follows:

- The initial assessment needs to be done in-person (i.e., hospital/clinic-based).
- Physical exercise can be done independently at home if the patient meets certain criteria.
- Education, monitoring and evaluation can be carried out in an offline-online combination depending on the patient's condition.
- Use of smartphone applications can help assess compliance, assist with interventions, and monitor safety or adverse effects,
- The implementation of telerehabilitation should be in accordance with the applicable laws and regulations.

Before the COVID-19 pandemic, there had been many studies looking at the effectiveness of home-based rehabilitation compared to center-based rehabilitation. One study reviewed 12 publications regarding telerehabilitation in patients with heart disease. It concluded that telerehabilitation is an effective and safe option for patients with heart disease (Batalik et al., 2020). Another study conducted a randomized controlled trial on patients with heart disease, comparing the intervention group that received teleconsultation assistance with conventional center-based rehabilitation. They found that the additional telerehabilitation program led to significant improvements in physical fitness and quality of life as compared to the control group (Frederix et al., 2015). Several similar studies have also shown the benefits of telerehabilitation compared to no telerehabilitation in patients with heart failure, post Myocardial Infarction (MCI), and Chronic Obstructive Pulmonary Disease (COPD) (Hwang et al., 2017; Tsai et al., 2017; Varnfield et al., 2014; Zanaboni et al., 2017).

**Figure 3:**
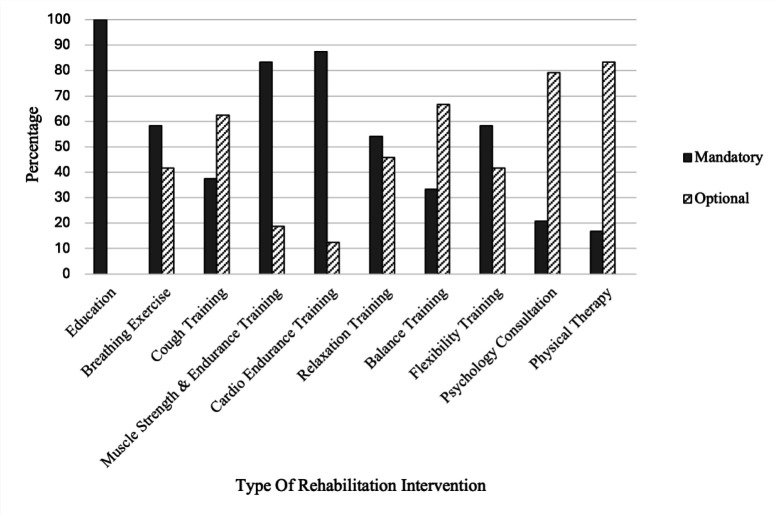
Opinion Regarding Rehabilitation Intervention for Long COVID (N = 24)

Long COVID has a very large variation of symptoms and degree of severity. As a result, it is necessary to adjust a prescribed rehabilitation program to the conditions found in the patient. This is in accordance with the panelists' response regarding the type of rehabilitation practice needed for patients with Long COVID. All panelists agreed upon three ‘mandatory’ types of rehabilitation practice in patients with Long COVID: provide education, perform cardiorespiratory endurance training, and perform muscle endurance and strength training. All panelists agreed upon two ‘optional’ types of intervention in patients with Long COVID: psychological consultation and physical therapy. There was no consensus for other types of interventions such as breathing exercise, effective cough training, relaxation training, balance training, and flexibility training. Figure 3 shows how the panelists viewed the necessity of various rehabilitation interventions, ranging from mandatory or optional (performed in certain conditions only).

We also explored the assessment needed to establish the prescription of rehabilitation interventions for patients with Long COVID. The panelists agreed that a comprehensive assessment is needed which includes anamnesis (i.e., recollection, as in a case history), physical examination, specific supporting and laboratory tests.

Table 2 shows the topics that must be explored during anamnesis. To anticipate the use of telerehabilitation, the ability of the patient to use digital technology becomes a necessary point to be explored during anamnesis.

**Table 2:** Information To Be Explored During Anamnesis

Information to be explored
- Current complaints (sequelae) - Daily routine of physical activity - Exercise habits - History of smoking - Past Medical History (pre COVID-19) - Past Medical History (post COVID-19) - List of medicines - Shortness of breath and oxygen supplementation requirement (at rest and during activity) - Support system (environment, family and socio-economic) - Exercise preferences - Patient expectations - Activity and participation limitations - Ability to use digital technology

**Figure 4:**
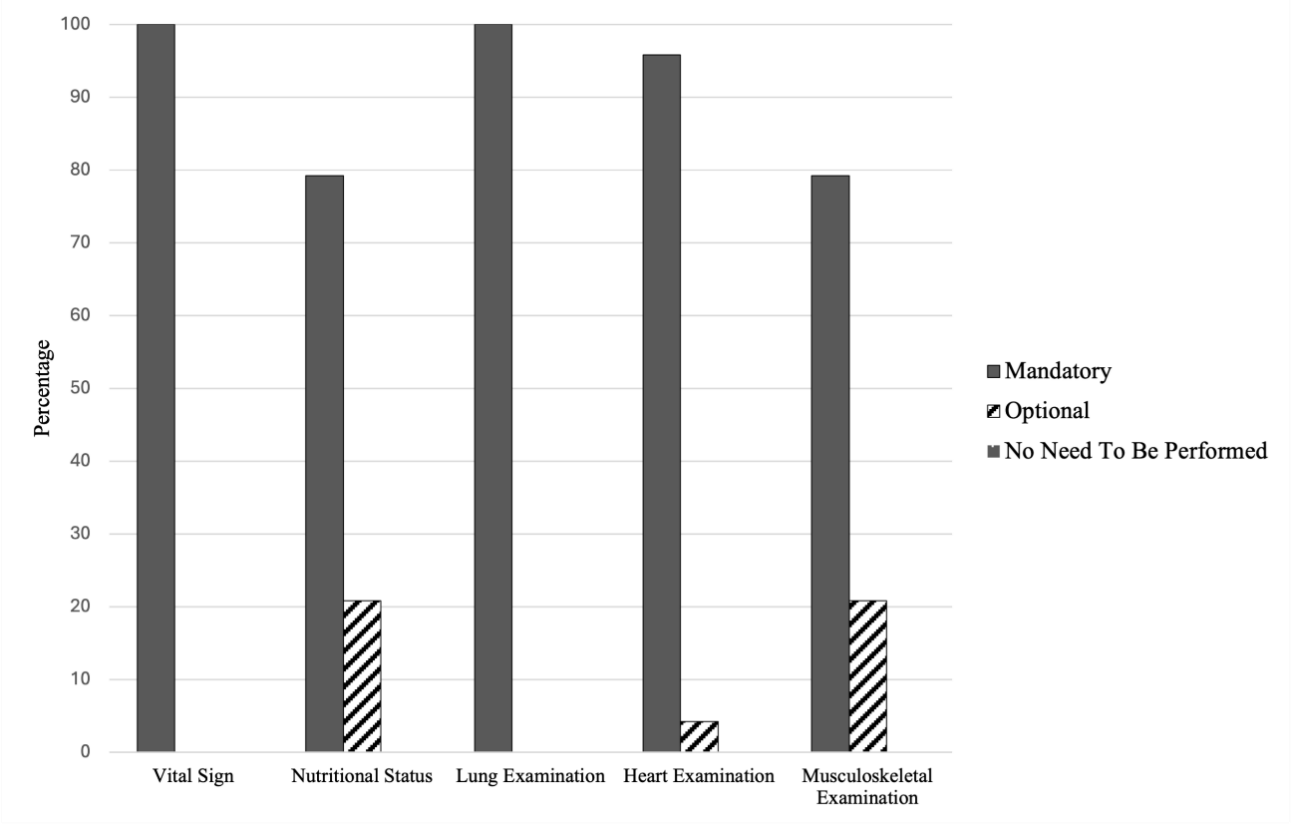
Types of Physical Examination to Determine Rehabilitation Intervention Long COVID

Figure 4 shows the opinions of the panelists regarding types of physical examination between 1 (no need to be performed); 2 (performed in certain condition), and 3 (must be performed) to determine rehabilitation intervention for the patient.

Next, we explore the specific supporting examinations that must be carried out to establish prescription of rehabilitation intervention. In the first round, authors collected inputs from the open-ended questions. In the second round of Delphi, from the entire list of tests collected, panelists were asked to choose between 1 (no need to be performed), 2 (performed in certain conditions/optional) and 3 (required to performed/mandatory). More than 80% of the panelists agreed that all examination on the list were mandatory to be carried out or should be carried out under certain conditions. We further explored the agreement between mandatory and optional. The content validity ratio (CVR) of each item was counted to determined agreement between examination that is mandatory (>= 0.417) and optional according to conditions of the patient (<= – 0.417). Table 3 shows the result of CVR of each item. Independency, fatigue, dyspnea, quality of life, chest expansion, and 6-minute walk test (6MWT) were items that were agreed to be mandatory. On the contrary, Cardiopulmonary Exercise Testing (CPET), Echocardiograph and Hand-held Dynamometer were agreed to be optional and carried out only if deemed necessary. No agreement was achieved for the rest of the items, meaning for several different reasons, around the same number of panelists was divided between the two choices. Considering the disagreement was regarding the choice between mandatory and optional, and not between ‘required’ or ‘no need to be performed', authors decided not to seek further agreement in these matters.

**Table 3:** Content Validity Ratio for Specific Supporting Examination

Specific supporting examination	Content Validity Ratio
Independency*	0.75
Cognition	0.25
Fatigue*	0.58
Dyspnea*	0.67
Depression and anxiety	0.33
Quality of sleep	0.08
Quality of life*	0.417
Chest expansion*	0.417
Spirometry	−0.167
Lung diffusion capacity	−0.167
6-minute walk test*	0.417
4-meter gait speed	−0.167
Cardiopulmonary Exercise Testing*	−0.5
Radiographic thorax X-Ray	0.17
Electrocardiograph	0.33
Echocardiograph*	−0.417
Hand-held Dynamometer*	−0.5
30 second Sit-to-stand	0.167

* *Note.* Show agreement from panelist that an exam mandatory (positive value) or optional (negative value).

For the laboratory test, panelists agreed that complete blood count is mandatory before determining a prescription for rehabilitation intervention. They further agreed that other laboratory tests were optional as per the condition of the patient.

All panelists in this study agreed that the initial screening must be carried out at the rehabilitation center (in-person services) to ensure the safety and validity of the examination results. This is in accordance with study by Tsai et al. (2017), which asked all participants to attend two visits at the hospital, before and immediately post intervention.

In one study of a telerehabilitation program in post-discharge COVID-19 patients, trained doctors performed the assessment via a home visit (Li et al., 2022). Due to limited number of trained doctors, the large geographic area, and heavy traffic in several areas in Indonesia, this method could not be applied to the current study.

One systematic review showed that in most telerehabilitation, all participants started the program under direct supervision of a specialist in a hospital center, followed by a remotely monitored telerehabilitation exercise (Batalik et al., 2020). Several studies gave all the participants a familiarization session either in-person at the hospital or during a home visit before telerehabilitation intervention was implemented (Avila et al., 2018; Bravo-Escobar et al., 2017; Hwang et al., 2017). This is in accordance with the findings of our study.

An offline adaptation phase with good training response was one of the prerequisites agreed by the all the panelists. In this study, each panelist was asked to list all items that they thought should be met before a patient could be signed to perform exercise independently at home to ensure the safety of the telerehabilitation program (Table 4).

**Table 4:** Input From the Panellists Regarding The Prerequisites For Physical Exercise Can Be Done Independently At Home By Patients With Long COVID

List of prerequisites
- Stable hemodynamic condition - No acute health problem - Accompanied by caregiver for frailty elderly, patients with severe disabilities, patients with uncontrolled comorbidity. - Adequate cognition - Patient and/or caregiver understands: how to assess vital signs how to assess exercise intensity symptoms and signs of not in the state to do physical exercise symptoms and signs to terminate physical exercise procedure if an adverse event occurs how to perform exercise movement correctly - Have the equipment and safe space for training - Adequate means of communication - Preceded by an adaptation phase offline and already observed to have a good training response - Digital literacy of the technology used

The ideal way to monitor home-based exercise sessions is real time monitoring using wearable devices. This method has been done in several studies for cardiac rehabilitation program (Fang et al., 2019; Maddison et al., 2019; Skobel et al., 2017). Previous studies reported that during telerehabilitation, the exercise intervention was monitored synchronously in real time either using electrocardiogram telemetry, wearable devices, heart rate sensor with chest strap fixing, or video conference (Avila et al., 2018; Bravo-Escobar et al., 2017; Hwang et al., 2017). One study in Indonesia showed that a 12-week exercise program for older people with dementia supervised by physiotherapists via synchronous online sessions with informal carer supervision at home, resulted in improvement of physical activity level, some aspects of function, health-related benefits of exercise, exercise enjoyment and quality of life. No falls or adverse events were reported in this study (Sari et al., 2023).

Delivering a telerehabilitation intervention is challenging, especially in a middle-income country such as Indonesia. A review of challenges to deliver telerehabilitation in low to middle income countries stated that human, organizational, and technical factors can all become obstacles. These factors usually overlap with one another, such as guidelines and laws on telemedicine (human and organizational); lack of digital knowledge and skills (human and technical); and lack of financing, governance, technical support, and training (organizational and technical)(Mohamad & Defi, 2023).

Due to limitations of funding and human resources, it will be difficult to provide wearable devices to each patient and to monitor their exercise sessions synchronously. Therefore, we propose asynchronous monitoring using several data that are input by the patient into a mobile Health (mHealth) application. One study also used this approach to monitor intensity and adherence to an exercise program (Varnfield et al., 2014). The authors were aware that for this approach to be successful, patients and/or caregivers must properly assess the parameters used for monitoring and be willing to honestly enter the subjective and objective data obtained from the self-measurement. These conditions were agreed upon by all the panelists as requirements to start a home exercise program (Table 4).

Panelists were asked which parameters are needed to monitor intensity and physiological response to exercise. They agreed that subjective feeling, peripheral oxygen saturation, peripheral pulse rate and rate of perceived exertion (RPE) using Borg Scale must be monitored pre and post exercise. A large cohort study showed that the Borg Scale (6-20) was strongly correlated with heart rate and blood lactate. Exercising at an RPE of 11-13 is recommended for less trained individuals, and an RPE of 13-15 may be recommended when more intense aerobic training is desired (Scherr et al., 2013).

It is not easy to determine the duration of a rehabilitation program, because individual needs vary. In addition, Long COVID is a “new” health condition with various symptoms and unclear pathophysiology. Nevertheless, understanding the course of change during rehabilitation may provide both clinician and patients with mindfulness regarding when they can expect changes to occur while participating in a rehabilitation program. Various studies have shown that regular physical exercise three times per week will improve cardiorespiratory endurance as early as the 3rd week, with improvements in vascular function from the 2nd week of exercise program (Gildea et al., 2021; Murias et al., 2010; Tinken et al., 2008). Guidelines from the British Thoracic Society stated that the acceptable duration of a pulmonary rehabilitation (PR) program is between six to twelve-weeks. The monitored outcomes of such programs are exercise capacity and health-related quality of life (HRQoL) (Bolton et al., 2013). Two studies that performed serial measurements of exercise capacity using 6MWT demonstrated a plateau of improvement at eight weeks of a 12-week PR program for patients with COPD (Rejbi et al., 2010; Solanes et al., 2009). Another study of a 12-week (24 sessions) PR program measured exercise capacity every two weeks by walks on a treadmill at a constant speed; the authors found the walk distance plateaued at week 10 (ZuWallack et al., 2006). Changes in HRQoL over the course of a PR program have been reported. In one study the greatest improvements were in the first two weeks (four sessions) (ZuWallack et al., 2006), and in another study at four weeks (12 sessions) (Solanes et al., 2009) with minimal later changes in either study. One study showed that a twice weekly, eight-week PR program (16 sessions) for moderate to very severe COPD patients can significantly improve the Endurance Shuttle Walk Test (ESWT), Six-Minute Walk Test (6MWT), St. George's Respiratory Questionnaire (SGRQ), and (COPD Assessment Test) CAT score as compared to baseline, with the greatest improvements occurring in the first four weeks of the program (Bishop et al., 2021). Taking all these data into account, the authors recommended a duration of 12 weeks for the rehabilitation program in Long COVID and asked the panelists for their opinions. All panelists agreed, with eight panelists adding that the program should be continued as needed.

For the evaluation at the program's end, the panelists agreed to re-evaluate all the assessments performed before the start of the program, with added emphasis to the importance of evaluating subjective complaints and obstacles in carrying out interventions, especially self-exercise at home, difficulties in using the mobile app, and compliance to the rehabilitation program in general.

## Conclusions

Large knowledge gaps persist regarding best practices in rehabilitation management for patients with Long COVID. One of the main reasons for this gap is that the pathophysiology of Long COVID is still not fully understood. Long COVID may affect survivors of COVID-19 at all disease severities. The symptoms of Long COVID vary greatly, and this has created obstacles to determining the most appropriate rehabilitation management.

In the current study we presented the results of a Delphi method involving 24 experts from different specialties and experience. Our findings provide guidelines for telerehabilitation intervention for patients with Long COVID in Indonesia or in a locale with similar conditions. Setting up a telerehabilitation intervention is challenging, therefore, further research is still needed to determine the feasibility and effectiveness of this guideline.

## Data Availability

The data used to support the findings of this study are available from the corresponding author upon request.

## Appendix A

### Questions used in Delphi Survey

#### Round 1

There are 43 statements/questions which are broken down into 4 parts. Please select an option for the statement items and write-down an explanation regarding your choices in the comment's column. Please provide detailed answers to all the questions.

**Table:**

No.	Statements	Comments Section
**Part 1. The Concept of Rehabilitation Intervention**		
	Rehabilitation interventions for Long COVID patients need to be provided:	
	To help resolve complaints. Strongly agree Agree Not sure Disagree Strongly disagree	
	By combining direct (offline) intervention and intervention via telemedicine (hereinafter referred to as the telerehabilitation model). Strongly agree Agree Not sure Disagree Strongly disagree	
	Tailored and adapted to the needs and conditions of each patient. Strongly agree Agree Not sure Disagree Strongly disagree	
	Comprehensively. Strongly agree Agree Not sure Disagree Strongly disagree	
	In your opinion, what aspects of health can be improved through rehabilitation interventions for Long COVID patients? Please state as much as possible, for example: fitness, pain complaints, etc.	
	In your opinion, what are the essential rehabilitation interventions given to COVID-19 survivors with residual symptoms? Please state as much as possible, for example: education, breathing exercises, etc.	
**Part 2. Initial Screening/Assessment**		
	Assessment/screening that needs to be carried out before starting and preparing a rehabilitation program prescription for Long COVID patient includes:	
	The ability to carry out daily activities independently. Strongly agree Agree Not sure Disagree Strongly disagree	
	The daily routine physical activity. Strongly agree Agree Not sure Disagree Strongly disagree	
	The exercise habits. Strongly agree Agree Not sure Disagree Strongly disagree	
	History of health disease suffered before contracting COVID-19. Strongly agree Agree Not sure Disagree Strongly disagree	
	History of health disease suffered contracting COVID-19. Strongly agree Agree Not sure Disagree Strongly disagree	
	List of medications currently taken. Strongly agree Agree Not sure Disagree Strongly disagree	
	Smoking habits. Strongly agree Agree Not sure Disagree Strongly disagree	
	Shortness of breath and oxygen requirements at rest and during activity. Strongly agree Agree Not sure Disagree Strongly disagree	
	Physical function. Strongly agree Agree Not sure Disagree Strongly disagree	
	Respiratory function. Strongly agree Agree Not sure Disagree Strongly disagree	
	Functional capacity test. Strongly agree Agree Not sure Disagree Strongly disagree	
	Psychological conditions (screening for depression and anxiety). Strongly agree Agree Not sure Disagree Strongly disagree	
	Cognitive function. Strongly agree Agree Not sure Disagree Strongly disagree	
	Electrocardiogram examination. Strongly agree Agree Not sure Disagree Strongly disagree	
	Echocardiogram examination. Strongly agree Agree Not sure Disagree Strongly disagree	
	Chest x-ray examination. Strongly agree Agree Not sure Disagree Strongly disagree	
	Complete peripheral blood laboratory test. Strongly agree Agree Not sure Disagree Strongly disagree	
	Blood coagulation laboratory test. Strongly agree Agree Not sure Disagree Strongly disagree	
	In your opinion, what other things need to be assessed to determine rehabilitation program prescriptions for Long COVID patients?	
**Part 3. Supervision/Monitoring**		
	Please express your opinion regarding the following parameters to be used as an instrument for monitoring and assessing rehabilitation interventions for Long COVID patients.	
	Oxygen saturation as a daily monitoring instrument pre and post exercise. Strongly agree Agree Not sure Disagree Strongly disagree	
	BORG scale as a daily monitoring instrument pre and post exercise. Strongly agree Agree Not sure Disagree Strongly disagree	
	Peripheral arterial pulse as a daily monitoring instrument pre and post exercise. Strongly agree Agree Not sure Disagree Strongly disagree	
	30 second Sit to Stand assessment as a weekly monitoring tool for rehabilitation interventions. Strongly agree Agree Not sure Disagree Strongly disagree	
	Assessment of the 6-minute walk test as a monthly monitoring tool for rehabilitation interventions. Strongly agree Agree Not sure Disagree Strongly disagree	
	Assessment of hand grip strength using a hand-held dynamometer as a monthly monitoring tool for rehabilitation interventions. Strongly agree Agree Not sure Disagree Strongly disagree	
	Quality of life assessment using the EQ-5D-5L questionnaire as a monthly monitoring tool for rehabilitation interventions. Strongly agree Agree Not sure Disagree Strongly disagree	
	In your opinion, what other instruments need to be used as parameters for monitoring and evaluating rehabilitation interventions for Long COVID patients?	
**Part 4. Telerehabilitation model**		
	Please share your opinion regarding the telerehabilitation model for Long COVID patients and provide an explanation of your choices in the comment's column.	
	An initial assessment which includes anamnesis, physical examination and functional examination needs to be carried out directly at the rehabilitation center. Strongly agree Agree Not sure Disagree Strongly disagree	
	A 12-week of rehabilitation intervention is sufficient. Strongly agree Agree Not sure Disagree Strongly disagree	
	Physical exercise can be done independently at home if meeting certain criteria. Strongly agree Agree Not sure Disagree Strongly disagree	
	In your opinion, what are the criteria needed for Long COVID patients to be able to carry out physical exercise independently at home? Please explain.	
	Education and mentoring can be done with synchronous telemedicine using a video conference platform. Strongly agree Agree Not sure Disagree Strongly disagree	
	Evaluation of rehabilitation programs can be carried out through a combination of in-person examinations at rehabilitation centers and video conference platforms. Strongly agree Agree Not sure Disagree Strongly disagree	
	The use of smartphone applications makes compliance monitoring easier. Strongly agree Agree Not sure Disagree Strongly disagree	
	The use of smartphone applications makes safety monitoring easier. Strongly agree Agree Not sure Disagree Strongly disagree	
	The use of smartphone application media makes supervising easier. Strongly agree Agree Not sure Disagree Strongly disagree	
	In your opinion, what other specific things needs to be of concern regarding the preparation of a telerehabilitation model for Long COVID patients? Please explain.	

## Appendix B

### Questions used in Delphi Survey

#### Round 2

**Table:**

Section 1 Benefits of rehabilitation interventions in Long COVID patients.	
Providing rehabilitation interventions to Long COVID patients can improve the patient's physical, psychological, and social aspects. The following is input from the panelists regarding aspects that can be improved through rehabilitation interventions: cardiovascular function (including fitness, exercise tolerance, heart rate) respiratory function (dyspnea, shortness of breath, expansion of the thorax) musculoskeletal function (muscle strength, muscle endurance, flexibility, coordination, balance) metabolic conditions pain (joints and muscles; headaches) independence quality of life functional capacity sleep quality psychological conditions (stress, depression, anxiety) tiredness/fatigue concentration disorders social life (participation)	
Referring to the list, do you agree that this list represents the improvements and benefits that can be achieved through rehabilitation interventions in Long COVID-19 patients? Agree Disagree	
If you disagree, please write down any health aspects that are not listed or that you disagree with and explain the reasons.	

Section 2. Types of rehabilitation interventions for Long COVID patients.	
Rehabilitation interventions for Long COVID patients must be provided in a tailor-made and comprehensive manner according to the patient's condition, needs and preferences. The following are inputs from the panelists regarding essential rehabilitation interventions that should be given to Long COVID patients. Please choose your assessment regarding each of the items:	
Education (includes education on lifestyle, activities, types of intervention, benefits of intervention, how to do physical exercise, intensity of physical exercise, how to assess the intensity of physical exercise, precaution in doing physical exercise, side effects of intervention, signs and symptoms that physical exercise should not be done, signs and symptoms that you should stop doing physical exercise, chart flow and management if an adverse event occurs, nutritional management) Compulsory (should always be given) Given in specific condition only No need to be given Other options: (please explain)	
Breathing exercises and expansion of the chest cavity Compulsory (should always be given) Given in specific condition only No need to be given Other options: (please explain)	
Effective cough exercises Compulsory (should always be given) Given in specific condition only No need to be given Other options: (please explain)	
Muscle endurance and strength training Compulsory (should always be given) Given in specific condition only No need to be given Other options: (please explain)	
Cardiorespiratory endurance training Compulsory (should always be given) Given in specific condition only No need to be given Other options: (please explain)	
Relaxation exercises Compulsory (should always be given) Given in specific condition only No need to be given Other options: (please explain)	
Balance training Compulsory (should always be given) Given in specific condition only No need to be given Other options: (please explain)	
Flexibility exercises Compulsory (should always be given) Given in specific condition only No need to be given Other options: (please explain)	
Psychiatric consultation Compulsory (should always be given) Given in specific condition only No need to be given Other options: (please explain)	
Specific physical therapy Compulsory (should always be given) Given in specific condition only No need to be given Other options: (please explain)	
Do you think there are any types of interventions that need to be added? Please write down and give your reasons.	
Are there any items with other options? Please include your comments regarding those items.	

Section 3. Combination of offline and online intervention	
Rehabilitation interventions for Long COVID patients can be carried out through a combination of offline meeting and online platform. Based on the results of the 1st round questionnaire analysis, here are several key points in relation to combine online-offline rehabilitation intervention: The initial assessment needs to be done offline Physical exercise can be done independently at home (if you meet certain criteria) Education, mentoring and evaluation can be carried out in a combination of offline and online (if certain criteria are met) Use of smartphone applications to help assess compliance, assist interventions, and monitor safety The implementation of telerehabilitation is in accordance with the local health regulations for telemedicine intervention	
Do you agree with the statements? Agree Disagree	
If you disagree, please write down any aspects that are not listed or that you disagree with and explain the reasons.	

Section 4. Criteria for independent physical exercise in Long-COVID patients.	
The following are inputs from the panelists regarding the criteria for allowing physical exercise can be done independently at home: Hemodynamic condition is stable Not in a condition of acute health problems There are companions for the elderly, patients with severe disabilities, patients with many uncontrolled comorbidities Have adequate cognition The patient/companion understands: how to assess vital signs how to assess exercise intensity symptoms and signs of not being able to do physical exercise symptoms and signs for termination of physical exercise flow of management if an adverse event occurs how to do exercise movements correctly Have equipment and a safe room for practice Have adequate communication tools It was preceded by an offline adaptation phase and was observed to have a good training response Digital literacy regarding the technology used	
If you look at the list, do you agree that this list represents the criteria needed for Long-COVID patients to be able to do physical exercise independently at home? Agree Disagree Other	
If you disagree, please write down other criteria that need to be added or that you disagree with and the reasons why.	

Section 5. Determining rehabilitation interventions prescriptions for Long-COVID patients.	
To determine rehabilitation interventions for Long-COVID patients, a comprehensive assessment is needed which includes history taking (anamnesis), physical examination, specific supporting examinations, and laboratory examinations.	
Anamnesis The following are the panelists' input regarding the information that needs to be explored in the anamnesis: Current complaints (sequelae) Daily routine physical activity Exercise habits Smoking history Past Disease History (pre COVID-19) Past Disease History (post COVID-19) List of medicines Ability to carry out daily activities independently Shortness of breath and need for oxygen (at rest and during activity) Support system (environment, family and socio-economic) Practice preferences Patient expectations Limitation of activities and participation Ability to use digital technology	
If you look at the list, do you agree that it represents the information that needs to be explored in Long-COVID patients to determine appropriate rehabilitation interventions? Agree Disagree	
If you do not agree, please write down what other information needs to be added or that you disagree with and the reasons why.	
Physical Examination The following is input from the panelists regarding the physical examination that needs to be carried out to determine appropriate rehabilitation interventions: Vital Signs Nutritional Status (BMI) Lung examination (inspection-palpation-auscultation) Heart examination (inspection-palpation-auscultation) Neuromusculoskeletal examination (posture, walking pattern, joint range of motion, muscle strength, balance, coordination, fine motor function)	
Please provide your opinion for the following physical examination:	
Vital Signs Compulsory (should always be performed) Performed in specific condition only No need to be performed Other options: (please explain)	
Nutritional Status (BMI) Compulsory (should always be performed) Performed in specific condition only No need to be performed Other options: (please explain)	
Lung examination Compulsory (should always be performed) Performed in specific condition only No need to be performed Other options: (please explain)	
Heart examination Compulsory (should always be performed) Performed in specific condition only No need to be performed Other options: (please explain)	
Neuromusculoskeletal examination Compulsory (should always be performed) Performed in specific condition only No need to be performed Other options: (please explain)	
Do you think there are other physical examinations that need to be added? Please include the reason.	
Are there items with other comment options? Please include your comments regarding this item.	
The following is input from the panelists regarding specific supporting examinations that need to be performed to determine appropriate rehabilitation intervention prescriptions for Long COVID patients. Please provide your further opinion for each examination item:	
Activity for Daily Living Compulsory (should always be performed) Performed in specific condition only No need to be performed Other options: (please explain)	
Cognitive function Compulsory (should always be performed) Performed in specific condition only No need to be performed Other options: (please explain)	
Fatigue level Compulsory (should always be performed) Performed in specific condition only No need to be performed Other options: (please explain)	
Dyspnea level Compulsory (should always be performed) Performed in specific condition only No need to be performed Other options: (please explain)	
Depression and anxiety level Compulsory (should always be performed) Performed in specific condition only No need to be performed Other options: (please explain)	
Sleep quality level Compulsory (should always be performed) Performed in specific condition only No need to be performed Other options: (please explain)	
Quality of life level Compulsory (should always be performed) Performed in specific condition only No need to be performed Other options: (please explain)	
Chest expansion function Compulsory (should always be performed) Performed in specific condition only No need to be performed Other options: (please explain)	
Spirometry Compulsory (should always be performed) Performed in specific condition only No need to be performed Other options: (please explain)	
Lung diffusion capacity Compulsory (should always be performed) Performed in specific condition only No need to be performed Other options: (please explain)	
Six Minute Walk Test Compulsory (should always be performed) Performed in specific condition only No need to be performed Other options: (please explain)	
4-m gait speed Compulsory (should always be performed) Performed in specific condition only No need to be performed Other options: (please explain)	
Cardiopulmonary exercise testing (CPET) Compulsory (should always be performed) Performed in specific condition only No need to be performed Other options: (please explain)	
Chest X-Ray Compulsory (should always be performed) Performed in specific condition only No need to be performed Other options: (please explain)	
Electrocardiogram Compulsory (should always be performed) Performed in specific condition only No need to be performed Other options: (please explain)	
Echocardiography Compulsory (should always be performed) Performed in specific condition only No need to be performed Other options: (please explain)	
Hand-held dynamometer Compulsory (should always be performed) Performed in specific condition only No need to be performed Other options: (please explain)	
30-s Sit-to-Stand Test Compulsory (should always be performed) Performed in specific condition only No need to be performed Other options: (please explain)	
Do you think there are other specific supporting physical examinations that need to be added? Please include the reason.	
Are there items with other comment options? Please include your comments regarding this item.	
Laboratory Examinations The following is input from the panelists regarding laboratory tests that need to be carried out to determine prescriptions for rehabilitation interventions. Please provide your further opinion for each examination item:	
Complete Blood Count Compulsory (should always be performed) Performed in specific condition only No need to be performed Other options: (please explain)	
D-dimer test Compulsory (should always be performed) Performed in specific condition only No need to be performed Other options: (please explain)	
Do you think there are other laboratory examinations that need to be added? Please include the reason.	
Are there items with other comment options? Please include your comments regarding this item.	

Section 6. Physical exercise monitoring	
The following is input from the panelists regarding parameters that can be used as monitoring tools before/during/immediately after physical exercise in Long-COVID patients. Please provide your further opinion for each of the following parameters:	
BORG scale (RPE) Compulsory (should always be performed) Performed in specific condition only No need to be performed Other options: (please explain)	
Talk test Compulsory (should always be performed) Performed in specific condition only No need to be performed Other options: (please explain)	
Subjective feeling (dizziness, dyspnea, severe pain, cold sweat, lethargic) Compulsory (should always be performed) Performed in specific condition only No need to be performed Other options: (please explain)	
Peripheral oxygen saturation Compulsory (should always be performed) Performed in specific condition only No need to be performed Other options: (please explain)	
Peripheral arterial pulse Compulsory (should always be performed) Performed in specific condition only No need to be performed Other options: (please explain)	
Do you think there are other instruments that need to be added for monitoring before/during/immediately after physical exercise? Please include the reason.	
Are there items with other comment options? Please include your comments regarding this item.	

Section 7. Evaluation of Long-COVID rehabilitation interventions	
The following is input from the panelists regarding things that need to be assessed to evaluate Long-COVID rehabilitation interventions.	
Please provide your further opinion for each of the following assessments:	
Subjective complaint/symptoms Compulsory (should always be assessed) Assessed in specific condition only No need to be assessed Other options: (please explain)	
Barriers/obstacles to carry out intervention Compulsory (should always be assessed) Assessed in specific condition only No need to be assessed Other options: (please explain)	
Compliance to intervention Compulsory (should always be assessed) Assessed in specific condition only No need to be assessed Other options: (please explain)	
Activity for Daily Living Compulsory (should always be assessed) Assessed in specific condition only No need to be assessed Other options: (please explain)	
Cognitive function Compulsory (should always be assessed) Assessed in specific condition only No need to be assessed Other options: (please explain)	
Fatigue level Compulsory (should always be assessed) Assessed in specific condition only No need to be assessed Other options: (please explain)	
Dyspnea level Compulsory (should always be assessed) Assessed in specific condition only No need to be assessed Other options: (please explain)	
Depression and anxiety level Compulsory (should always be assessed) Assessed in specific condition only No need to be assessed Other options: (please explain)	
Sleep quality level Compulsory (should always be assessed) Assessed in specific condition only No need to be assessed Other options: (please explain)	
Quality of life level Compulsory (should always be assessed) Assessed in specific condition only No need to be assessed Other options: (please explain)	
Chest expansion function Compulsory (should always be assessed) Assessed in specific condition only No need to be assessed Other options: (please explain)	
Spirometry Compulsory (should always be assessed) Assessed in specific condition only No need to be assessed Other options: (please explain)	
Lung diffusion capacity Compulsory (should always be assessed) Assessed in specific condition only No need to be assessed Other options: (please explain)	
Six Minute Walk Test Compulsory (should always be assessed) Assessed in specific condition only No need to be assessed Other options: (please explain)	
4-m gait speed Compulsory (should always be assessed) Assessed in specific condition only No need to be assessed Other options: (please explain)	
Cardiopulmonary exercise testing (CPET) Compulsory (should always be assessed) Assessed in specific condition only No need to be assessed Other options: (please explain)	
Chest X-Ray Compulsory (should always be assessed) Assessed in specific condition only No need to be assessed Other options: (please explain)	
Electrocardiogram Compulsory (should always be assessed) Assessed in specific condition only No need to be assessed Other options: (please explain)	
Echocardiography Compulsory (should always be assessed) Assessed in specific condition only No need to be assessed Other options: (please explain)	
Hand-held dynamometer Compulsory (should always be assessed) Assessed in specific condition only No need to be assessed Other options: (please explain)	
30-s Sit-to-Stand Test Compulsory (should always be assessed) Assessed in specific condition only No need to be assessed Other options: (please explain)	
D-dimer test Compulsory (should always be assessed) Assessed in specific condition only No need to be assessed Other options: (please explain)	
Do you think there are other aspects that need to be added to the evaluation? Please include the reason.	
Are there items with other comment options? Please include your comments regarding this item.	

Section 8. Duration of Long-COVID rehabilitation intervention	
Long COVID is something new. Research regarding Long COVID is still ongoing. Based on research for Long COVID patients at Persahabatan National Respiratory Referral Hospital, symptoms persisted for 1 month in 54.3% of sufferers and persisted for 1 to 6 months in 43% of sufferers. The symptoms of Long COVID are highly variable and the underlying pathophysiology is not yet fully understood. Various internal and external factors are thought to influence symptom improvement. Rehabilitation interventions by mainly providing prescribed physical exercise are expected to help improve the Long COVID symptoms by improving cardiorespiratory fitness, muscle strength and endurance, the circulation function, the immune function, and reducing pain. Various literature shows that the impact of regular and adequate physical exercise on physical fitness, maximum oxygen uptake, and increased muscle strength is generally visible in the 12th week.	
Based on these considerations, what do you think about the 12-week duration of rehabilitation intervention for Long COVID patients? Agree (already adequate) Agree (with possibility to be extended) Disagree	
If you disagree, please write down the reasons and opinions.	
